# Association between anthropometric measures and abdominal wall thickness in patients with obesity: A cross-sectional CT study

**DOI:** 10.1371/journal.pone.0354274

**Published:** 2026-07-30

**Authors:** Prasit Mahawongkajit, Porrawat Rodsa, Saritphat Orrapin

**Affiliations:** 1 Department of Surgery, Faculty of Medicine, Thammasat University, Pathum Thani, Thailand; 2 Thammasat University Hospital, Thammasat University, Pathum Thani, Thailand; University of Montenegro-Faculty of Medicine, MONTENEGRO

## Abstract

**Background:**

Abdominal wall thickness (AWT) is a critical determinant of safe abdominal access in patients with obesity, yet it is not routinely assessed preoperatively. Simple, bedside methods for evaluating factors associated with AWT are lacking. This study aimed to evaluate the association between readily available anthropometric measurements and AWT across multiple surgically relevant abdominal regions.

**Methods:**

In this prospective cross-sectional study, 105 patients with obesity (body mass index [BMI] ≥30 kg/m^2^) who underwent computed tomography (CT) imaging were included. AWT was measured at nine predefined anatomical points corresponding to commonly used surgical landmarks. Anthropometric parameters, including BMI, neck circumference, mid-upper arm circumference, and waist circumference, were recorded. Correlation analyses were performed to assess the associations between anthropometric measurements and CT-measured AWT.

**Results:**

BMI demonstrated a moderate and consistent positive association with AWT across all anatomical regions (rs = 0.34–0.55, all p < 0.001). Waist circumference and mid-upper arm circumference showed moderate associations, whereas neck circumference demonstrated weak and inconsistent associations. AWT varied across anatomical regions, with greater thickness observed in the lower abdomen. Female patients had significantly greater AWT at several sites. These findings highlight regional variability in AWT and its association with anthropometric measurements.

**Conclusions:**

Anthropometric measurements, particularly BMI, were significantly associated with AWT across multiple abdominal regions in patients with obesity. These findings provide additional insight into regional abdominal wall variability and may support future development of bedside assessment tools for abdominal access planning. Further studies are needed to develop and validate predictive models for clinical application.

## Introduction

The global prevalence of obesity continues to rise, including in Thailand, largely driven by lifestyle changes such as increased consumption of high-calorie diets and reduced physical activity [1,2]. As a result, surgeons are increasingly encountering patients with obesity who require abdominal surgical interventions. Minimally invasive surgery (MIS) has become widely adopted across a broad range of procedures and offers several advantages over conventional open surgery, including smaller incisions, reduced risk of wound infection, less postoperative pain, and faster recovery [3,4].

In patients with obesity, laparoscopic surgery is generally preferred when feasible, as it may reduce postoperative complications associated with open surgery, particularly wound infection and wound dehiscence. However, increased abdominal wall thickness in this population presents a significant technical challenge. Abdominal wall thickness is a key factor influencing the difficulty of abdominal access and may affect the selection of instrument length, including trocars and Veress needles. It may also influence the risk of access-related complications during abdominal procedures [5–8].

Beyond elective laparoscopic surgery, safe and reliable abdominal access is also essential in emergency and percutaneous procedures. In trauma settings, particularly in cases of penetrating abdominal injury, rapid access to the abdominal cavity is critical but may be technically challenging in patients with obesity. Similarly, procedures such as abdominal paracentesis, feeding tube placement, and percutaneous drainage may be affected by increased abdominal wall thickness. Knowledge of abdominal wall anatomy and thickness may therefore facilitate procedural planning across a range of clinical settings [5–8].

Previous studies have suggested that simple anthropometric measurements may reflect body fat distribution and abdominal wall characteristics. These measurements are easy to perform, cost-effective, and do not require specialized equipment [9–12]. Parameters such as neck circumference, mid-upper arm circumference, waist circumference, and body mass index (BMI) have been widely used as indicators of obesity and central adiposity. Prior studies have demonstrated that these measures are associated with overall adiposity and may correlate with anatomical features of the anterior abdominal wall on computed tomography (CT), including abdominal wall thickness [13,14]. However, data remain limited regarding the association between anthropometric measurements and abdominal wall thickness at multiple surgically relevant anatomical landmarks in individuals with obesity.

Despite its clinical importance, abdominal wall thickness is not routinely assessed preoperatively, and practical bedside methods for evaluating factors associated with this parameter are lacking. Therefore, this study aimed to evaluate the association between anthropometric measurements and abdominal wall thickness across nine predefined abdominal regions in patients with obesity. The findings may provide additional insight into regional abdominal wall variability and support future development of bedside assessment approaches for abdominal access planning.

## Materials and methods

This study was conducted as a single-center, prospective cross-sectional study at Thammasat University Hospital. Patients aged 15–80 years with obesity (BMI ≥ 30 kg/m^2^) who underwent CT whole abdomen imaging for any indication were eligible for inclusion. Patients were excluded if they had abdominal wall abnormalities (e.g., abdominal wall hernia, including umbilical hernia, or colostomy), previous abdominal surgery, neck abnormalities (e.g., neck mass or tracheostomy), upper arm abnormalities (e.g., deformity or large mass), clinically significant weight change (defined as ≥5% body weight loss within one month), CT imaging performed more than 30 days prior to anthropometric assessment, or the presence of ascites (e.g., due to chronic liver cirrhosis or advanced intra-abdominal malignancy).

The study protocol was approved by the Human Research Ethics Committee of Thammasat University (Medicine) (Project No. MTU-EC-ES-1-119/67; COA No. 195/2024). The study was conducted in accordance with the Declaration of Helsinki and Good Clinical Practice guidelines. Participant recruitment was conducted prospectively between 05/08/2024 and 04/08/2025. Written informed consent was obtained from all participants prior to enrollment. For participants younger than 18 years of age, written informed consent was obtained from their parents or legal guardians.

Sample size was calculated to detect a correlation between BMI and anterior abdominal wall thickness (AWT). Based on preliminary data from 10 patients with BMI ≥ 30 kg/m^2^ who underwent CT imaging, the expected correlation coefficient was 0.2778. Using a two-tailed test with a significance level of 0.05 and a statistical power of 80%, the required sample size was estimated to be 96.22. Accordingly, a minimum sample size of 97 participants was considered adequate for this study.

**Fig 1:**
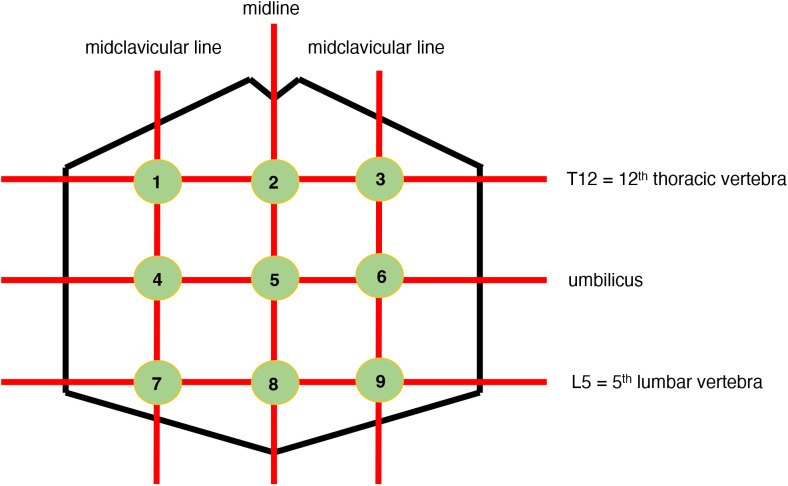
Schematic illustration of the nine predefined anatomical points on the abdominal wall used for measuring abdominal wall thickness (AWT) on computed tomography images. These points correspond to commonly used surgical landmarks for abdominal access in laparoscopic and percutaneous procedures.

Clinical data were collected from the hospital electronic medical record system, outpatient surgical clinic records, and the CT unit of the Department of Radiology. The recorded variables included age, sex, comorbidities, body weight, height, BMI, indication for CT examination, neck circumference (cm), mid-upper arm circumference (cm), and waist circumference (cm). AWT, measured in millimeters (mm), was assessed using CT images at nine predefined points on the abdominal wall corresponding to commonly used surgical landmarks (Fig 1). All CT measurements were performed by an experienced radiologist.

The primary outcome was the association between anthropometric parameters (BMI, neck circumference, mid-upper arm circumference, and waist circumference) and CT-measured AWT at each anatomical site. Secondary outcomes included comparison of AWT across different abdominal regions and evaluation of the associations between anthropometric measurements and regional AWT. Anthropometric measurements were obtained using standardized techniques. Neck circumference was measured with the patient in the Frankfort horizontal position at the midpoint of the neck between the clavicle and the mandible. Mid-upper arm circumference was measured at the midpoint between the acromion and the olecranon process. Waist circumference was measured at the level of the umbilicus. All anthropometric measurements were performed by trained personnel using a non-stretchable measuring tape.

Continuous variables were expressed as mean ± standard deviation (SD) for normally distributed data or median with interquartile range (IQR) for non-normally distributed data. Group comparisons were performed using the unpaired t-test or the Mann–Whitney U test, as appropriate. The relationship between anthropometric parameters and CT-measured AWT was assessed using correlation analysis. Normality was evaluated using the Kolmogorov–Smirnov test and the Doornik–Hansen omnibus test. Pearson correlation was applied for normally distributed variables with bivariate normality, whereas Spearman rank correlation was used for non-parametric data. Scatter plots with fitted linear regression lines and 95% confidence intervals were constructed to illustrate the relationships between anthropometric variables and AWT at each measurement site. All statistical analyses were performed using STATA/MP version 18.0 for Mac (StataCorp LP, College Station, TX, USA), and a two-sided p-value <0.05 was considered statistically significant. The study was reported in accordance with the STROBE guidelines for cross-sectional studies.

## Results

A total of 105 participants were included in the analysis, exceeding the calculated minimum sample size. The study population consisted of 52 males and 53 females, with a mean age of 55.0 ± 13.7 years. Baseline characteristics are summarized in Table 1. Only one participant (0.95%) was younger than 18 years of age (17 years old); all remaining participants were adults. Male participants had significantly higher body weight and height than female participants (93.46 ± 14.67 vs. 81.96 ± 10.76 kg, p < 0.001; 167.62 ± 8.39 vs. 155.17 ± 6.30 cm, p < 0.001, respectively), whereas BMI was comparable between the two groups (33.14 ± 3.27 vs. 34.00 ± 3.61 kg/m^2^, p = 0.200). Neck circumference and mid-upper arm circumference were significantly greater in males, whereas waist circumference did not differ significantly between sexes.

**Table 1:** Patient characteristics with computed tomography measured abdominal wall thickness and anthropometric parameters.

Variables	Male (n = 52)	Female (n = 53)	Total (N = 105)	*P-value*
Age (years) mean ± SD	52.94 ± 14.05	57.02 ± 13.22	55 ± 13.72	0.1289
Body weight (kg) mean ± SD	93.46 ± 14.67	81.96 ± 10.76	87.66 ± 14.03	<0.0001
Height (cm) mean ± SD	167.62 ± 8.39	155.17 ± 6.3	161.33 ± 9.66	<0.0001
BMI (kg/m^2^) mean ± SD	33.14 ± 3.27	34 ± 3.61	33.57 ± 3.46	0.2005
Arm circumference (cm) mean ± SD	34.19 ± 4.17	36.93 ± 4.48	35.57 ± 4.53	0.0016
Waist circumference (cm) mean ± SD	109.5 ± 10.17	109.47 ± 11.72	109.49 ± 10.93	0.9895
Neck circumference (cm) mean ± SD	42.27 ± 3.63	38.58 ± 3.13	40.41 ± 3.84	<0.0001
Abdominal wall thickness (mm)				
Area1 mean ± SD	34.63 ± 6.44	39.23 ± 6.93	36.61 ± 7.05	0.0006
Area2 mean ± SD	26.06 ± 9.05	33.58 ± 7.84	29.59 ± 9.23	<0.0001
Area3 mean ± SD	35.38 ± 6.72	38.23 ± 6.67	36.5 ± 6.81	0.0319
Area4 mean ± SD	40.58 ± 11	43.19 ± 7.58	41.54 ± 9.48	0.1608
Area5 mean ± SD	23.31 ± 9.15	26.75 ± 7.06	24.86 ± 8.3	0.0334
Area6 mean ± SD	39.67 ± 9.89	41.87 ± 7.87	40.45 ± 8.95	0.2119
Area7 mean ± SD	44.42 ± 10.89	47.45 ± 9.7	45.59 ± 10.37	0.1357
Area8 mean ± SD	37.46 ± 10.89	44.04 ± 10.6	40.47 ± 11.19	0.0022
Area9 mean ± SD	43.1 ± 9.89	45.91 ± 8.48	44.18 ± 9.27	0.1215

BMI, body mass index; cm, centimeters; mm, millimeters; iqr, interquartile range; kg, kilogram; m, meters; SD, standard deviation.

CT-measured AWT varied across anatomical regions and between sexes. Female participants demonstrated significantly greater AWT at several measurement points, including Area 1 (39.23 ± 6.93 vs. 34.63 ± 6.44 mm, p = 0.0006), Area 2 (33.58 ± 7.84 vs. 26.06 ± 9.05 mm, p < 0.001), Area 3 (38.23 ± 6.67 vs. 35.38 ± 6.72 mm, p = 0.0319), Area 5 (26.75 ± 7.06 vs. 23.31 ± 9.15 mm, p = 0.0334), and Area 8 (44.04 ± 10.60 vs. 37.46 ± 10.89 mm, p = 0.0022). No statistically significant differences were observed at the remaining sites. Overall, AWT demonstrated regional variability, with thicker measurements generally observed in the lower abdominal regions.

Correlation analysis demonstrated that BMI was significantly associated with AWT across all nine anatomical regions (Spearman’s rho ranging from 0.34 to 0.55, all p < 0.001). Waist circumference also showed significant positive associations with AWT at most sites (rs = 0.23–0.49, p < 0.05), particularly in the lower abdominal regions. Mid-upper arm circumference demonstrated moderate positive associations with AWT (rs = 0.25–0.43, p < 0.05), although these associations were not statistically significant at all anatomical sites. In contrast, neck circumference showed weaker and less consistent associations with AWT, reaching statistical significance only at selected regions (rs = 0.19–0.21, p < 0.05). Age demonstrated weak negative associations with AWT at several anatomical sites, particularly in the middle abdominal regions. The correlations between anthropometric parameters and AWT at each anatomical site are summarized in Table 2.

**Table 2:** The association between anthropometric parameters and computed tomography measured abdominal wall thickness.

Anthropometric parameters related with abdominal wall thickness	Correlation coefficient (Spearman’s rho (r_s_))^*,**^								
	Area 1	Area 2	Area 3	Area 4	Area 5	Area 6	Area 7	Area 8	Area 9
Age (years) (mean ± SD)	−0.07 P = 0.47	0.01 P = 0.91	−0.19 P = 0.04	−0.33 P < 0.001	−0.24 P = 0.01	−0.38 P < 0.001	−0.18 P = 0.05	−0.24 P = 0.01	−0.17 P = 0.06
BMI (kg/m^2^) (mean ± SD)	0.55 P < 0.001	0.35 P < 0.001	0.45 P < 0.001	0.41 P < 0.001	0.34 P < 0.001	0.39 P < 0.001	0.51 P < 0.001	0.41 P < 0.001	0.47 P < 0.001
Neck circumference (cm) (mean ± SD)	0.01 P = 0.88	−0.05 P = 0.56	0.04 P = 0.66	0.12 P = 0.21	0.01 P = 0.93	0.21 P = 0.02	0.19 P = 0.04	0.06 P = 0.49	0.21 P = 0.02
Mid-upper arm circumference (cm) (mean ± SD)	0.43 P < 0.001	0.27 P = 0.01	0.35 P < 0.001	0.33 P < 0.001	0.18 P = 0.06	0.30 P < 0.001	0.41 P < 0.001	0.25 P = 0.01	0.43 P < 0.001
Waist circumference (cm) (mean ± SD)	0.44 P < 0.001	0.23 P = 0.01	0.35 P < 0.001	0.24 P = 0.01	0.16 P = 0.08	0.25 P = 0.01	0.49 P < 0.001	0.32 P < 0.001	0.47 P < 0.001

BMI, body mass index; cm, centimeters; mm, millimeters; kg, kilogram; m, meters; SD, standard deviation

*One-sample Kolmogorov-Smirnov test against theoretical normal distribution, the Spearman Rank correlation coefficient test for non-parametric test.

**Against the bivariate normality by Doornik–Hansen omnibus tests, the Spearman Rank correlation coefficient test was assessed.

**Fig 2:**
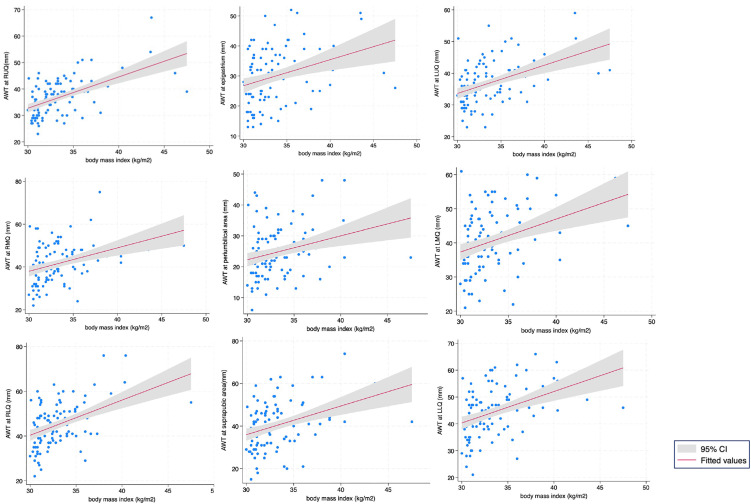
Scatter plots with fitted linear regression lines demonstrating the relationship between body mass index and abdominal wall thickness across nine predefined abdominal regions. Right upper quadrant (RUQ), epigastrium, left upper quadrant (LUQ), right middle quadrant (RMQ), periumbilical region, left middle quadrant (LMQ), right lower quadrant (RLQ), suprapubic region, and left lower quadrant (LLQ).

**Fig 3:**
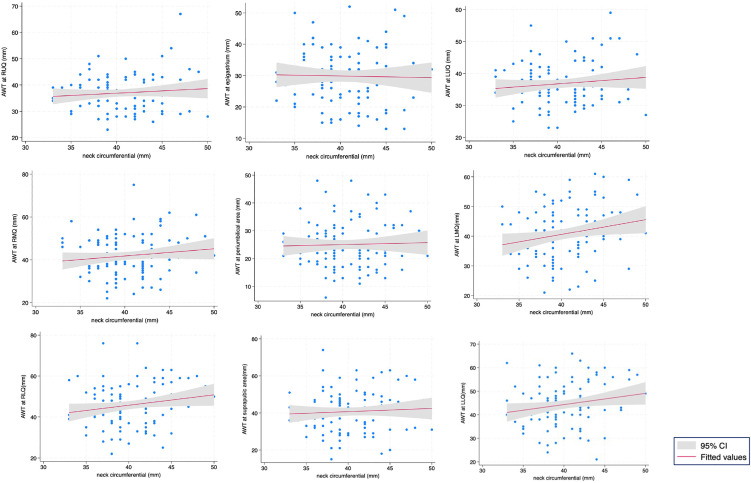
Scatter plots with fitted linear regression lines demonstrating the relationship between neck circumference and abdominal wall thickness at nine predefined abdominal regions. Right upper quadrant (RUQ), epigastrium, left upper quadrant (LUQ), right middle quadrant (RMQ), periumbilical region, left middle quadrant (LMQ), right lower quadrant (RLQ), suprapubic region, and left lower quadrant (LLQ).

**Fig 4:**
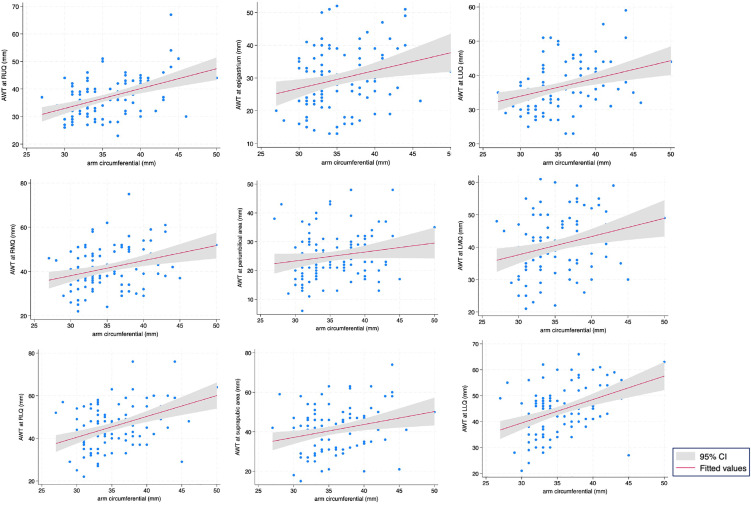
Scatter plots with fitted linear regression lines demonstrating the relationship between mid-upper arm circumference and abdominal wall thickness at nine predefined abdominal regions. Right upper quadrant (RUQ), epigastrium, left upper quadrant (LUQ), right middle quadrant (RMQ), periumbilical region, left middle quadrant (LMQ), right lower quadrant (RLQ), suprapubic region, and left lower quadrant (LLQ).

**Fig 5:**
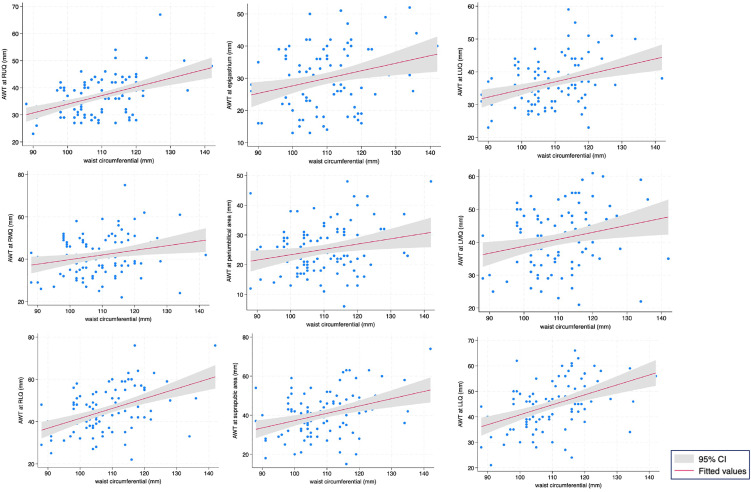
Scatter plots with fitted linear regression lines demonstrating the relationship between waist circumference and abdominal wall thickness at nine predefined abdominal regions. Right upper quadrant (RUQ), epigastrium, left upper quadrant (LUQ), right middle quadrant (RMQ), periumbilical region, left middle quadrant (LMQ), right lower quadrant (RLQ), suprapubic region, and left lower quadrant (LLQ).

Scatter plots with fitted linear regression lines further illustrated the relationships between anthropometric parameters and AWT across the nine predefined anatomical regions (Fig 2, Fig 3, Fig 4 and Fig 5). BMI demonstrated consistent linear associations with AWT across all anatomical regions, whereas waist circumference and mid-upper arm circumference showed associations at most sites. Neck circumference demonstrated relatively weak and inconsistent associations with AWT.

## Discussion

This study demonstrates that simple anthropometric measurements, particularly BMI, waist circumference, and mid-upper arm circumference, are significantly associated with AWT across multiple anatomically relevant regions in patients with obesity. Only one participant was younger than 18 years of age; therefore, the findings primarily reflect adults with obesity and should be interpreted accordingly. Among the anthropometric parameters evaluated, BMI was significantly associated with AWT across all measurement sites, whereas the other anthropometric measures demonstrated associations at selected anatomical regions.

Abdominal access represents a critical step in both laparoscopic and percutaneous procedures. In patients with obesity, increased AWT may increase the technical difficulty of trocar insertion and Veress needle placement, and may also be associated with a higher risk of access-related complications, including failed entry, visceral injury, and vascular injury. The present findings suggest that readily available anthropometric measurements are associated with regional AWT and may provide useful information during preprocedural assessment. However, because this study evaluated associations rather than predictive performance, these measurements should not be considered substitutes for imaging when accurate assessment of AWT is required.

In addition to BMI, waist circumference and mid-upper arm circumference demonstrated moderate associations with AWT, suggesting that these parameters may reflect regional adiposity and abdominal wall characteristics. Waist circumference, which reflects central obesity, showed associations at most lower abdominal regions commonly used for laparoscopic access and percutaneous procedures. Mid-upper arm circumference also demonstrated consistent, although weaker, associations with AWT. In contrast, neck circumference showed limited and inconsistent associations with AWT, suggesting that it may have limited value as an anthropometric marker of abdominal wall thickness.

The regional variability in AWT observed in this study has important clinical relevance. Certain anatomical regions, particularly in the lower abdomen, demonstrated greater wall thickness. These findings are consistent with previous imaging-based studies reporting heterogeneous distribution of abdominal wall thickness and fat deposition and provide additional information regarding regional anatomical variation in patients with obesity. The observed sex differences in AWT despite comparable BMI may be explained by differences in body fat distribution. Females generally have greater subcutaneous fat accumulation, whereas males tend to accumulate more visceral fat [15]. Because CT-measured AWT primarily reflects subcutaneous tissue thickness, these physiological differences may account for the greater AWT observed in female participants despite similar BMI values. These findings also highlight that BMI alone does not fully capture regional fat distribution.

Beyond elective laparoscopic surgery, these findings may also be relevant to emergency and percutaneous procedures. In trauma settings, rapid abdominal access may be technically challenging in patients with obesity. Likewise, procedures such as abdominal paracentesis, gastrostomy tube placement, and percutaneous drainage may be influenced by abdominal wall thickness. Because anthropometric measurements are simple, inexpensive, and readily obtainable at the bedside, they may complement clinical assessment when imaging is unavailable. However, further studies are required to determine whether their use improves procedural planning or clinical outcomes.

This study has several strengths. It prospectively evaluated AWT at nine predefined anatomical points corresponding to commonly used surgical landmarks, providing a comprehensive assessment of regional variation. The use of CT-based measurements ensured accurate and objective quantification of abdominal wall thickness. In addition, the inclusion of multiple anthropometric parameters enabled comparison of their associations with regional AWT in a clinically relevant population.

Several limitations should also be acknowledged. First, this was a single-center study, which may limit the generalizability of the findings. Second, although CT provides accurate measurement of AWT, it is not routinely performed solely for this purpose in clinical practice. Third, this study evaluated associations rather than developing predictive models or clinically applicable cutoff values. Fourth, multiple correlation analyses were performed across nine anatomical sites without adjustment for multiplicity; therefore, findings with borderline statistical significance should be interpreted with caution. Finally, clinical outcomes, such as access-related complications, were not evaluated, and therefore the clinical utility of anthropometric measurements for abdominal access requires further investigation.

Future studies should develop and validate multivariable predictive models incorporating anthropometric parameters to estimate AWT and evaluate their performance in independent cohorts. Studies examining whether such models improve procedural planning and clinical outcomes would further establish their potential clinical utility.

In conclusion, simple anthropometric measurements were significantly associated with abdominal wall thickness in patients with obesity. BMI was significantly associated with AWT across all anatomical regions evaluated. These findings improve understanding of regional abdominal wall anatomy and may support the future development of bedside assessment tools. Further validation studies are required before anthropometric measurements can be used for clinical decision-making.

## Supporting information

S1 Dataset Anonymized minimal dataset underlying the findings of this study. The dataset contains de-identified participant demographic characteristics, anthropometric measurements (body weight, height, body mass index, neck circumference, mid-upper arm circumference, and waist circumference), and CT-measured abdominal wall thickness at the nine predefined anatomical sites included in the analyses. (XLSX)

## Abbreviations

AWT: abdominal wall thickness
BMI: body mass index
CT: computed tomography
IQR: interquartile range
LLQ: left lower quadrant
LMQ: left middle quadrant
LUQ: left upper quadrant
MIS: minimally invasive surgery
RMQ: right middle quadrant
RLQ: right lower quadrant
RUQ: right upper quadrant
SD: standard deviation
